# Non‐Invasive Tumor‐Naïve Minimal Residual Disease Detection of Liver Cancer by Incorporating Circulating Tumor DNA Features and Alpha‐Fetoprotein: A Prospective Study

**DOI:** 10.1002/cam4.70511

**Published:** 2024-12-20

**Authors:** Qingqi Ren, Shiyong Li, Guolin Zhong, Yunfei Li, Dao‐Ling Huang, Liangliang Zhang, Yumin Feng, Guanghui Long, Mao Mao

**Affiliations:** ^1^ Department of Hepatopancreatobiliary Surgery Peking University Shenzhen Hospital Shenzhen China; ^2^ Research & Development, SeekIn Inc. Shenzhen China; ^3^ Clinical Laboratories Shenyou Bio Zhengzhou China; ^4^ Research & Development, SeekIn Inc. San Diego California USA; ^5^ Yonsei Song‐Dang Institute for Cancer Research Yonsei University Seoul Korea

**Keywords:** AFP, CNA, fragment size, liver cancer, MRD, sWGS

## Abstract

**Background and Purpose:**

Liver cancer has a high recurrence rate of 50%~70% for early‐stage patients. Minimal residual disease (MRD) is strongly linked to liver cancer early recurrence. Identifying MRD through reliable prognostic biomarkers, such as circulating tumor DNA (ctDNA), could significantly benefit these patients by enabling timely intervention and improved outcomes.

**Materials and Methods:**

A prospective study enrolled 32 liver cancer patients undergoing radical surgery. Peripheral blood samples (8 mL) were collected before and after surgery. In this study, we expanded upon our previously developed multi‐omics assay, initially designed for liver cancer early detection by calculating a cancer signal score (P_HCC_), to determine the MRD status (named SeekInCure). This process integrated protein tumor marker alpha‐fetoprotein (AFP) and cancer genomic hallmarks, copy number aberration (CNA) and fragment size (FS).

**Results:**

Of the enrolled patients, 78.1% were in early stages, and before surgery, 87.5% of patients had successfully detected the cancer signal in blood. After radical surgery, 23 patients were MRD‐negative, exhibiting better overall survival compared to the MRD‐positive patients (*n* = 9, *p* < 0.01). Patients maintaining undetectable cancer signals pre‐ and post‐surgery showed 100% survival, conversely, those keeping with detectable signals had a 55.6% mortality rate.

**Conclusion:**

This prospective study highlights the prognostic value of ctDNA‐based tumor‐naïve MRD detection through a multi‐omics assay in early‐stage liver cancer patients.

## 1 Introduction

Liver cancer is the sixth most common cancer and the third leading cause of cancer‐related mortality worldwide in 2020 [1, 2]. In China, where the hepatitis B infection rate is high, liver cancer is the fourth in cancer morbidity and the second in cancer mortality [3]. Primary liver cancer includes hepatocellular carcinoma (HCC) (comprising 75%–85% of cases), which is an aggressive disease for its poor prognosis and high risk of recurrence with a 1‐year survival < 50% and 5‐year survival of around 10% [4, 5, 6]. In particular, the recurrence rate is 50%–70% for patients with early‐stage liver cancer based on the Barcelona Clinic Liver Cancer (BCLC) staging systems [7, 8, 9]. Obviously, tumor recurrence poses a significant challenge to improving survival outcomes for liver cancer patients.

There has been strong evidence that the early and late recurrence of tumors including liver cancer lies in quite different mechanisms [8, 10, 11, 12]. Specifically, minimal residual disease (MRD) can be the potential major cause of early recurrence within 2 years, and recurrence occurring after 2 years may be mainly due to de novo tumors in the microenvironment predisposed to carcinogenesis [9, 13, 14, 15]. MRD reflects the microscopic neoplastic substance remaining in the body after a curative treatment but is undetectable in traditional surveillance methods such as alpha‐fetoprotein (AFP) levels and imaging techniques [16, 17, 18].

Currently, there are two major strategies for detecting MRD based on circulating tumor DNA (ctDNA), tumor‐informed and tumor‐naïve approaches [19, 20, 21]. Tumor‐informed MRD method identifies tumor‐derived somatic mutations in tumor tissues of cancer patients after surgery, followed by MRD monitoring in plasma [22, 23]. Therefore, tumor‐informed MRD detection assays are highly patient‐specific and often necessitate customized gene panels [19]. By contrast, tumor‐naïve MRD detection assays rely solely on cell‐free DNA (cfDNA) with fixed genetic features, making them a non‐invasive, easy‐to‐operate, and cost‐effective alternative [24]. While tumor‐naïve MRD detection assays may be less sensitive compared to tumor‐informed assays, they offer a more comprehensive genetic profile by overcoming the spatial limitations of tissue biopsies [24]. Additionally, their performance can be enhanced by incorporating other biomarkers [25, 26]. Consequently, tumor‐naïve assays have attracted growing attention in multiple cancers such as lung, colon, breast, esophagus, and stomach cancers [11, 27, 28, 29]. It was suggested that MRD detection may be utilized in monitoring recurrence [30, 31, 32], guiding adjuvant treatment [22, 33, 34], and driving clinical trials [23, 35, 36]. Recently, plasma‐only ctDNA was first studied in liver cancer to design a panel of 13 MRD monitoring genes by targeted next‐generation sequencing (NGS) for predicting the risk of early postoperative recurrence [37].

Previously, our group developed a blood‐based multi‐omics assay for liver cancer early detection, which integrated genomics hallmarks such as copy number aberration (CNA) and fragment size (FS) from shallow whole genome sequencing (sWGS) of cfDNA with the protein marker AFP [38]. Here, we expanded the application of the multi‐omics method to detect cancer signals in the blood of liver cancer patients following radical surgery. This prospective study affirmed that plasma‐based MRD detection demonstrated the prognostic value in survival end points, suggesting limited benefits of cancer‐related treatment for postoperative patients with negative MRD results. These findings highlight the potential clinical value of our method, a sufficient and cost‐effective blood‐based tumor‐naïve multidimensional assay named SeekInCure, for MRD detection in patients after surgery, aiding in the prediction of those who may not require additional treatment.

## 2 Materials and Methods

### 2.1 Patients and Study Design

Liver cancer patients who underwent radical surgery at the Peking University Shenzhen Hospital from May 2018 to February 2022 were recruited. The inclusion criteria were as follows: (a) liver cancer confirmed by pathology, (b) no preoperative antitumor treatment, and (c) R0 resection. The exclusion criterion was as follows: presence or history of malignancies in extrahepatic organs. All participants provided written informed consent upon enrollment, and the study protocol shared the same ethical approval as the previous study from the independent ethics committee of Peking University Shenzhen Hospital [38].

### 2.2 Sample Process and AFP Quantification

Peripheral blood samples (8 mL) were obtained from each patient prior to the initiation of any treatment for cancer including surgery and at a median of 7.5 days after surgery (range: 5–46 days) using Cell‐Free DNA BCT blood collection tubes (Streck, Omaha, USA). The blood samples were centrifuged at 1600×*g* for 10 min at 4°C, and the resulting supernatants were collected and further centrifuged at 16,000×*g* for 10 min at 4°C. Plasma was then divided into two tubes: one containing 350 μL plasma for quantifying AFP levels, and the remaining plasma (at least 2 mL) was used for cfDNA extraction. The process of AFP quantification was described in the previous study [38].

### 2.3 cfDNA Extraction, Library Construction, and Sequencing

The extraction of cfDNA from the remaining plasma and library construction were consistent with our previously published work [38]. Prepared libraries were sequenced on a NovaSeq system (Illumina, San Diego, CA) using PE150 for WGS, resulting in the generation of approximately 10 GB of raw data (~3× coverage).

### 2.4 CNA Analysis

After cutting adapters and removing low‐quality reads, the resulting clean reads were aligned to human genome (https://hgdownload.soe.ucsc.edu/goldenPath/hg38/bigZips/) using BWA‐MEM software (v0.7.17) with default parameters. Based on the sequencing alignment results, CNA was analyzed using the method described in our previously published work [38].

### 2.5 FS Analysis

We have expanded our FS analysis method to encompass not only the global short fragment ratio P150 [38], representing the proportion of short fragments (< 150 bp) to the total number of fragments (30–250 bp), but also the regional short‐to‐long ratio. As described in the previous publications [39, 40, 41], the regional short‐to‐long ratio involved dividing the human genome into non‐overlapping bins of 5 Mb and calculating the ratio of short (100–150 bp) to long (151–220 bp) fragments within each bin. To reduce dimensionality, principal component analysis (PCA) was utilized. Subsequently, the top 10 principal component features were selected, collectively accounting for 85% of the variance in the fragmentation profiles across training samples. These training samples were obtained from our previous study, which included 76 liver cancer patients and 247 healthy individuals [38]. Finally, the global short fragment ratio P150 was combined with the 10 principal component features to calculate the final FS value using a GBM model based on the same training cohort [39, 40, 41].

### 2.6 Cancer Signal Score (P_HCC_ ) Calculation and MRD Status

The final artificial intelligence (AI) model was developed based on the CNA and FS values, in conjunction with the AFP level to calculate the cancer signal score, defined as P_HCC_ value. This was accomplished using the same methodology and training cohort as detailed in our previously published study [38]. Utilizing the approach outlined in the same published study, which achieved a specificity of 98.0%, the cutoff value of P_HCC_ was determined to be 0.475. A P_HCC_ value of a preoperative blood sample exceeding this threshold indicated the cancer signal was detectable in the sample, defined as molecular tumor burden positive (MTB+); conversely, values below indicated MTB−. Similar to the MTB status determination, the status of MRD in postoperative blood samples was determined using the same threshold. The same model and cutoff value were utilized to generate the P_HCC_ value and determine the status of preoperative and postoperative blood samples obtained from the patients enrolled in this prospective study.

### 2.7 Statistical Analysis

Statistical analysis was performed using R software (v4.0.1). Survival analysis including the Kaplan–Meier survival function and Cox regression for hazard ratios (HRs) was calculated by the survminer package (v.0.4.9).

## 3 Results

### 3.1 Demographic Characteristics and Schematic of the Multi‐Omics Approach

A total of 32 liver patients undergoing radical surgery were prospectively recruited eligibly in this study, which included 28 HCC patients, two intrahepatic cholangiocarcinoma (ICC) patients, and two combined hepatocellular cholangiocarcinoma (cHCC‐ICC) patients. Detailed clinical information for each patient is summarized in Table 1. The median age at diagnosis was 52 years old (range: 30–77 years). 84.4% of patients were male. As for cancer stage, most patients had early stage, including 65.6% stage I and 12.5% stage II, whereas 21.9% patients had stage III. 90.6% of patients were infected with hepatitis B virus (HBV).

**TABLE 1:** Patient demographics and clinical information.

Characteristics	Patients (*n* = 32)
Age at diagnosis, years	
Median (range)	52 (30–77)
≥ 55	19 (59.4%)
< 55	13 (40.6%)
Gender, *n* (%)	
Female	5 (15.6%)
Male	27 (84.4%)
Histological subtype, *n* (%)	
HCC	28 (87.5%)
ICC	2 (6.3%)
cHCC‐ICC	2 (6.3%)
Stage of disease, *n* (%)	
I	21 (65.6%)
II	4 (12.5%)
III	7 (21.9%)
AFP, IU/mL	
Median	1666
≥ 20	24 (75.0%)
< 20	8 (25.0%)
HBV, *n* (%)	
Yes	29 (90.6%)
No	3 (9.4%)

Abbreviations: AFP, alpha‐fetoprotein; cHCC‐ICC, combined hepatocellular cholangiocarcinoma; HBV, hepatitis B virus; HCC, hepatocellular carcinoma; ICC, intrahepatic cholangiocarcinoma.

**FIGURE 1:**
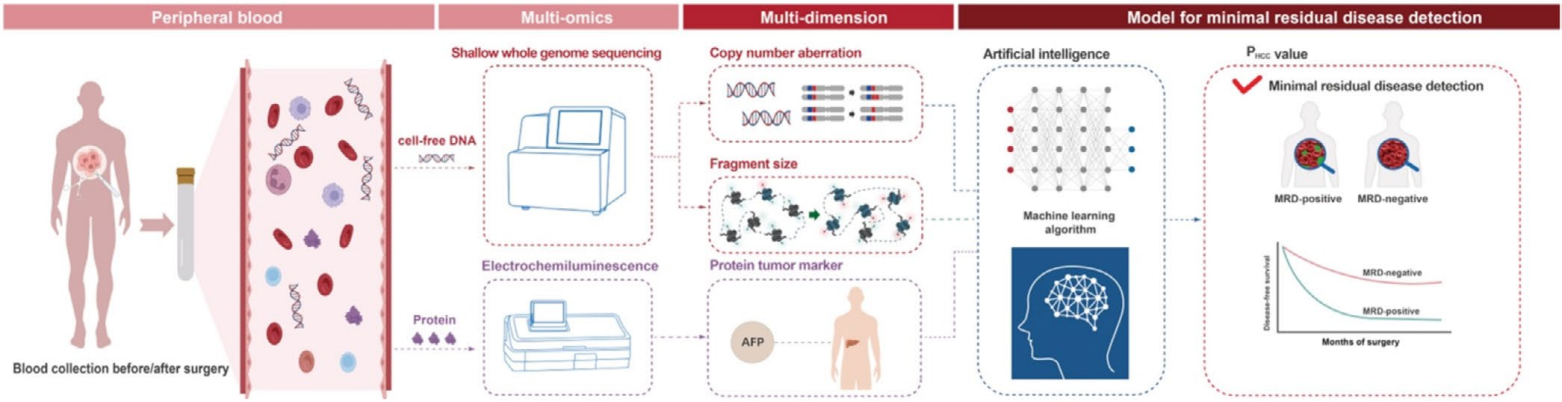
Illustration of the multi‐omics approach to calculate P_HCC_ value and determine MRD status. Schematic illustration of SeekInCure in MRD detection for liver cancer patients. A tube of 8 mL peripheral blood of cancer patients was collected before and after radical surgery, respectively, followed by the measurements of CNA and FS by sWGS, and protein tumor markers such as AFP. These measurements of each patient were then fed into machine learning algorithms to obtain the baseline and MRD status. Based on follow‐up data, the probabilities of survival of patients were estimated. MRD, minimal residual disease.

The method to detect MRD in early‐stage liver cancer patients using SeekInCure assay is schematically illustrated in Figure 1. In brief, a tube of peripheral blood (8 mL) was collected from each cancer patient before and after radical surgery. Subsequently, the genomic hallmarks such as CNA and FS were measured based on shallow whole genome sequencing, and the protein tumor marker AFP was quantified by the Roche Cobas e411. An AI model was employed to calculate the value of P_HCC_, which was then utilized to determine the MTB and MRD statuses.

### 3.2 Performance of Updating FS Method

In our previously published study, only the global ratio of short fragments (P150) was utilized in FS analysis to differentiate between liver cancer patients and healthy individuals. Healthy subjects exhibited a mean P150 value of 14.2. For example, in the pre‐surgery blood sample of patient P09, the P150 value was 12.9, and the distribution of global FS in P09 closely resembled the average distribution observed in the healthy individuals (*n* = 247) (Figure S1A). However, a significant deviation from the normal pattern was observed in the FS regional (5‐Mb bins) short‐to‐long ratio, particularly evident in chromosomes 4, 8, and 13. Compared to the background average distribution of the regional short‐to‐long ratio in healthy individuals, the regional short‐to‐long ratio in chr4 of P09 was lower than the average regional ratio in the healthy individuals, while the regional short‐to‐long ratios in chr8 and chr13 were notably higher than the average regional ratio in the healthy individuals (Figure S1B). Consequently, we updated the FS method to incorporate the regional short‐to‐long ratio with the global short ratio P150 using the GBM model. This predicted the FS value of P09 as 0.867, notably higher than the median FS value of 0.179 in the healthy individuals. Upon assessment in the training cohort, the updated FS method exhibited an increased AUC for cancer detection, rising significantly from 0.828 with the published FS method P150 to 0.931 (*p* < 0.001, Figure S1C).

### 3.3 MRD Status Was Determined by the CNA, FS, and AFP

**FIGURE 2:**
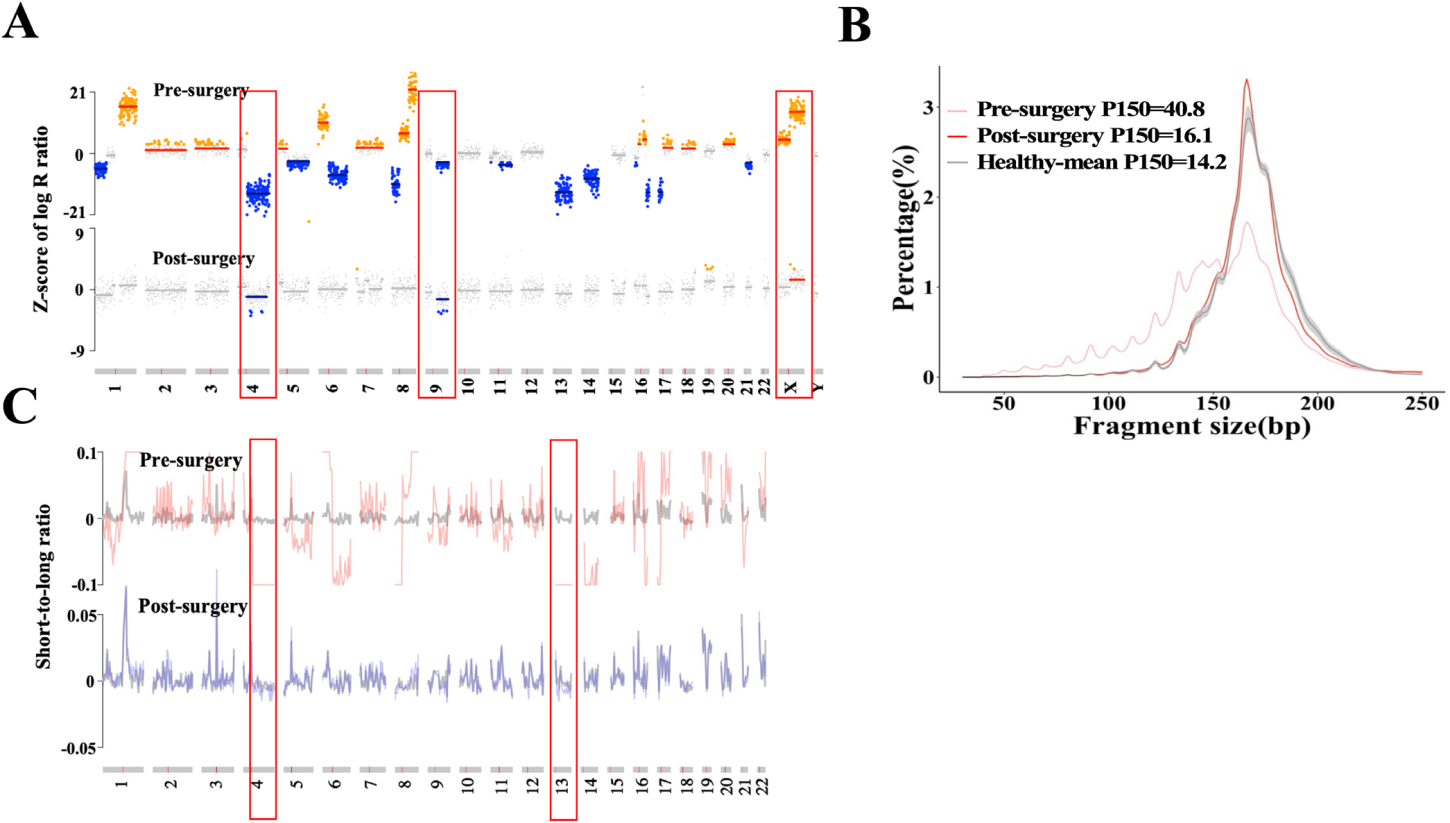
The dynamic changes of copy number aberration (CNA) and fragment size (FS) in pre‐ and post‐surgery blood samples of a patient were illustrated. (A) CNA analysis of peripheral blood samples from Patient P05 before and after surgery. (B) Shallow whole genome sequencing was utilized to infer the global size distribution of cfDNA fragments from patient P05's pre‐ and post‐surgery blood samples. The gray region represented the central distribution (the 25th and 75th percentiles) of fragment size frequencies obtained from 247 healthy individuals, while the black line depicts the mean value of fragment size across the healthy individuals. (C) Regional (5‐Mb) short‐to‐long ratio in patient P05's pre‐ and post‐surgery blood samples. The gray line represents the background distribution of regional short‐to‐long ratios, calculated as the mean value across 5‐Mb regions from the healthy individuals.

Patient P05, a 64‐year‐old male with HCC at stage III, exhibited multiple CNAs with varying amplitudes in the pre‐surgery blood sample. Although most CNAs disappeared in the post‐surgery sample, deletions in chromosomes 4 and 9q, as well as amplification in chromosome X, persisted in post‐surgery plasma samples, indicating detectable residual cancer signal in P05's post‐surgery samples (Figure 2A).

Simultaneously, the final FS value in the pre‐surgery sample was 0.987, characterized by a high proportion of short reads (P150 = 40.8) and a significant bias of regional short‐to‐long ratio in multiple chromosomes, such as chr4, chr8, chr13, and others (Figure 2B,C). Following radical surgery, the P150 value decreased to 16.1 in the post‐surgery sample, slightly higher than the mean value observed in the healthy individuals (*n* = 247). Additionally, the regional short‐to‐long ratio in chr4 and chr13 showed a slight bias toward the background distribution of healthy individuals. After combining them, the FS value decreased to 0.621, indicating the persistence of a positive cancer signal in the post‐surgery sample.

AFP levels decreased from 39.9 to 8.6 ng/mL. Moreover, leveraging AI to integrate CNA, FS, and AFP data and calculate the cancer score (P_HCC_), the final P_HCC_ value decreased from 0.999 to 0.871, higher than the cutoff value of 0.475, indicating a positive MRD status. Unfortunately, patient P05 died 100 days after radical surgery, which was consistent with MRD status and the dynamic change of P_HCC_ before and after surgery. This demo case underscored the predictive value of MRD status for prognosis.

**FIGURE 3:**
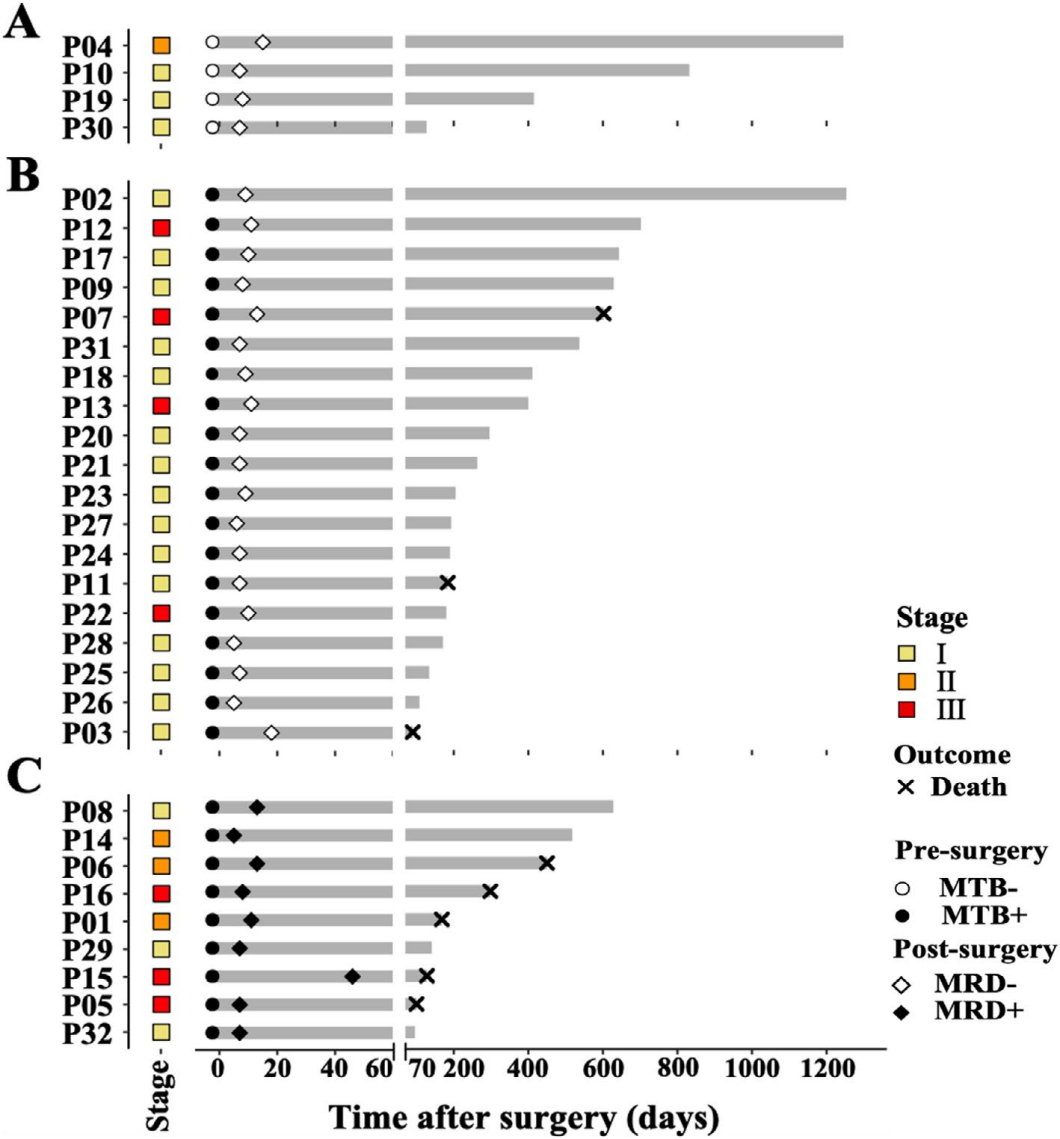
The swimmer plot depicted the change in ctDNA levels from pre‐surgery to post‐surgery, clinical factors, and survival. P01–P32 indicated the patient numbers of all 32 liver cancer patients. The squares with different colors, empty/solid circles, empty/solid rhombuses, and crosses correspond to the subgroups of stages, MTB−/+, MRD−/+, and death, respectively. MTB, molecular tumor burden; MRD, molecular residual disease.

To illustrate the dynamic changes in P_HCC_ value and clinical outcomes for individual patients, the P_HCC_ values from pre‐ and post‐surgery samples were expressed in a binary format (positive or negative) using a swimmer plot (Figure 3). The horizontal axis depicted the duration of follow‐up in days after surgery, while each patient was illustrated by a separate vertical bar. Patients were initially grouped based on their cancer signal status in the pre‐surgery samples, with negative cases shown in Figure 3A. Subsequently, positive patients were further grouped based on their cancer signal status in the post‐surgery samples, with negative and positive cases shown in Figure 3B,C, respectively.

### 3.4 MRD Status Is Associated With Survival End Points

**FIGURE 4:**
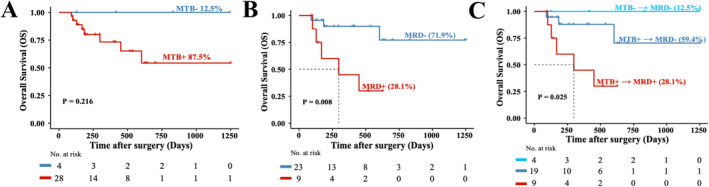
The ctDNA‐based MRD status was predictive of survival end point in postoperative patients with liver cancer. Kaplan–Meier estimates for OS stratified by cancer signal status (undetectable vs detectable) in pre‐surgery blood samples (A), post‐surgery blood samples (B), and the dynamic change of status before and after surgery (C). MTB+/− denoted detectable/undetectable in preoperative liver cancer patients; MRD+/− denoted detectable/undetectable in postoperative liver cancer patients; MTB, molecular tumor burden; MRD, molecular residual disease.

As illustrated in Figure 4A, the P_HCC_ values in 87.5% (28/32) of patients' pre‐surgery blood samples exceeded the cutoff value, indicating MTB+ status. Among these, 28.6% (8/28) of liver cancer patients died within the 602 days following radical surgery. Conversely, 12.5% (4/32) of liver patients with P_HCC_ value below the cutoff value (MTB‐), indicating undetectable cancer signals, exhibited a 100% overall survival (OS) rate. The obtained *p*‐value of 0.216 from the log‐rank test exceeded the significance threshold of 0.05, possibly due to the small sample size in the MTB‐ group (*n* = 4).

After radical surgery, there was a notable reduction in P_HCC_ value. Only 28.1% (9/32) of post‐surgery samples exhibited P_HCC_ values exceeding the cutoff value, indicative of detectable cancer signals, thus defined as MRD+ status. Conversely, 71.9% (23/32) of post‐surgery samples had P_HCC_ values below the cutoff, termed MRD− status. The Kaplan–Meier analysis revealed a significant discrepancy in OS between MRD+ and MRD− patients, with a log‐rank test yielding a *p*‐value of less than 0.01 (Figure 4B). Notably, MRD+ patients displayed a substantially reduced median OS of 298 days compared to MRD− patients, whose median OS was not reached.

We further compared the dynamic changes in P_HCC_ value before and after surgery, 12.5% (4/32) of patients kept with undetectable cancer signals in both before and after surgery blood samples exhibited a 100% survival rate in a median follow‐up time of 624 days (range: 127–1245 days). Among the patients (28/32) with evident cancer signals in pre‐surgery samples, 67.9% (19/28) showed undetectable cancer signals in post‐surgery samples, with only 15.8% (3/19) experiencing mortality in a median follow‐up time of 234 days (range: 90–1253 days). Conversely, 32.1% (9/28) of patients maintained detectable cancer signals in the before and after surgery samples, with a mortality rate of 55.6% (5/9) in a median follow‐up time of 168 days (range: 96–628 days). These findings underscored the strong association between MRD status and clinical survival end points, emphasizing the clinical significance of dynamic P_HCC_ changes in pre‐ and post‐surgery samples. The log‐rank test further revealed a statistically significant difference in overall survival among the three groups (*p* < 0.05, Figure 4C).

### 3.5 MRD Status Was the Most Significant Prognostic Factor Associated With OS

**FIGURE 5:**
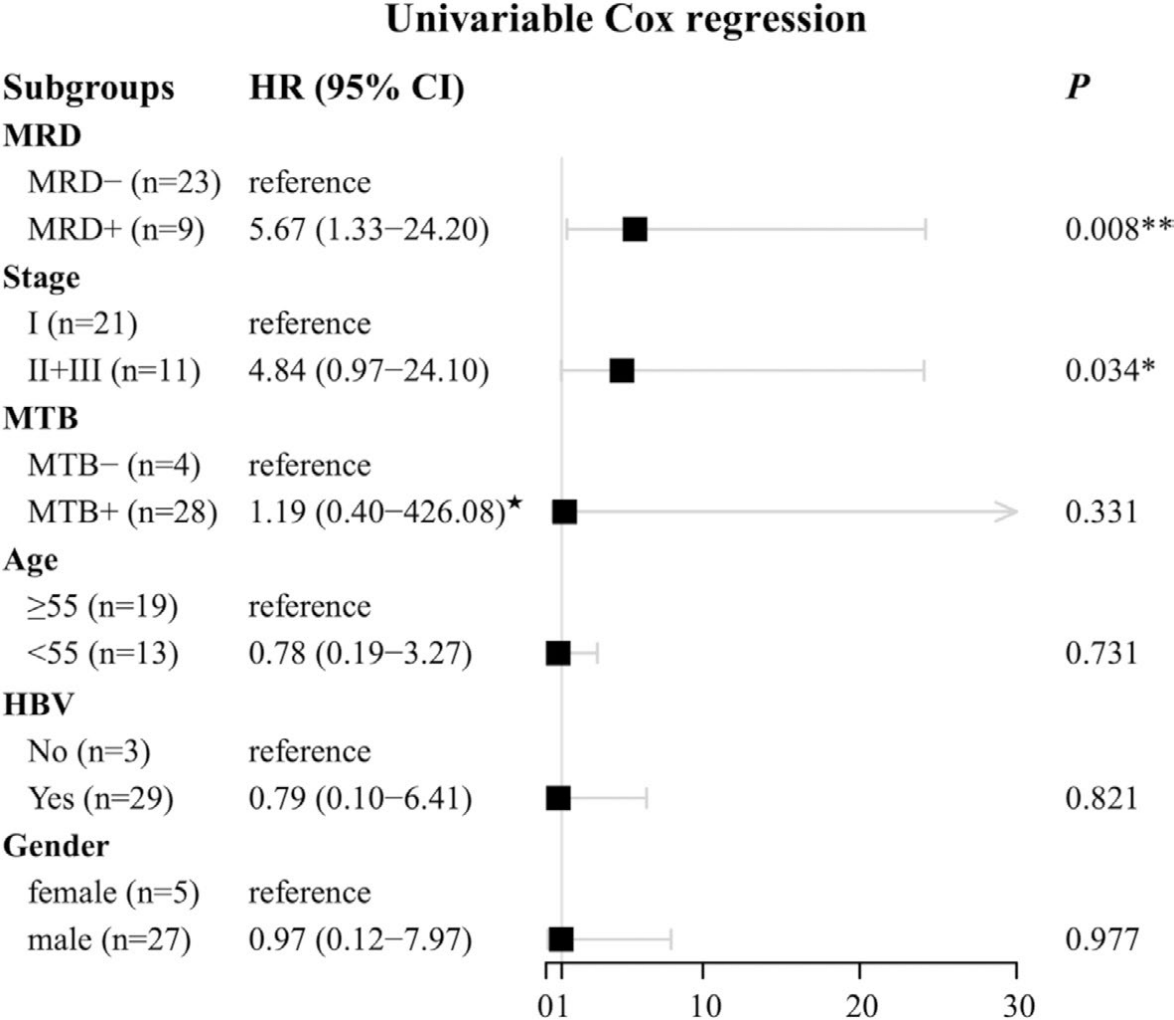
Forest plot depicting hazard ratios and 95% confidence intervals from univariate analysis for OS in liver cancer. Various factors (clinical factors, MTB and MRD statuses) and their association with OS were estimated by the univariable Cox proportional‐hazards model. Points and error bars indicate HR and 95% CI, respectively. HR, hazard ratio; MTB, molecular tumor burden; MRD, minimal residual disease; HBV, hepatitis B virus. *p < 0.05 (log‐rank test), **p < 0.005 (log‐rank test). ★ The “coxphf” package using the Firth‐penalized Cox regression maximum likelihood bias reduction method was used to estimate the coefficients and confidence intervals in the MTB group due to no events.

Univariable Cox regression analysis in Figure 5 revealed that MRD and stage were independent prognostic predictors of the OS. MRD had the strongest independent association with the OS (HR 5.67, 95% confidence interval (CI) 1.33–24.20; *p* = 0.008), followed by stage (HR 4.84, 95% CI 0.97–24.10; *p* = 0.034). It is noted that the HR value and confidence intervals in the MTB group were estimated using the Firth‐penalized Cox regression maximum likelihood bias reduction method due to no events in the MTB− group [42].

## 4 Discussion

In this prospective study, we evaluated the prognostic value of ctDNA‐based MRD status detection using a multi‐omics assay (SeekInCure), which incorporated genomic features such as CNA and FS with the protein feature AFP. This approach represented a tumor‐naïve strategy, profiling ctDNA without relying on the mutation status in tumor tissue. This study demonstrated the clinical value of MRD status in predicting the prognosis of liver cancer patients following radical surgery.

The blood‐based multi‐omics assay, initially developed for liver cancer early detection, was expanded for MRD detection. In our prior study, this assay achieved a sensitivity of 75.0% at a specificity of 98.0%. In this prospective cohort study, SeekInCure detected 87.4% of liver cancer patients at the same specificity level in the pre‐surgery blood samples. Notably, all patients were newly diagnosed and untreated, with 78.1% of them classified as early stages (I/II), reaffirming the high sensitivity of our assay for detecting cancer signals and validating its robustness.

MRD is widely considered a critical factor contributing to early postoperative relapse, and the use of ctDNA, employing either tumor‐informed or tumor‐naïve approach, has demonstrated efficacy in detecting MRD across various tumor types [27, 28, 29]. The published studies on MRD detection based on ctDNA in liver cancer patients were mainly using the tumor‐informed strategy [14, 26]. These studies demonstrated that the tumor‐informed strategy was feasible for monitoring MRD, but its broader application is hampered by inherent limitations, including heterogeneity of liver cancer, inability to detect emerging mutations, limited access to tumor tissue, and high associated costs. In contrast, our SeekInCure assay for MRD detection capitalizes on several advantages. By incorporating the protein tumor marker AFP, which retained the benefits of current clinical methods, and employing sWGS to detect the whole genomic CNA and FS profiles, we aimed to detect the genomic‐level changes and to reduce processing time and costs, particularly for analyzing cancer tissue. This approach enhances its versatility, accessibility, and robustness for all liver cancer patients, including those with ICC and cHCC‐ICC. Although AFP has demonstrated value in monitoring ICC recurrence, its utility is limited to a small subset of ICC patients [43, 44]. In our study, AFP levels were normal in two ICC and two cHCC‐ICC cases. However, sWGS analyses of cfDNA successfully detected cancer signals in one ICC and two cHCC‐ICC cases preoperatively. Following radical surgery, these cases transitioned to MRD‐negative status, aligning with clinical outcomes. In this prospective study, we further found patients kept with undetectable cancer signals in both before and after surgery samples exhibited a 100% survival rate in a median follow‐up time of 624 days. Conversely, patients who retained detectable signals in the before and after surgery samples experienced a mortality rate of 55.6% over a median follow‐up period of 168 days. These findings not only highlighted the clinical predictive value of MRD detection in survival end points but also emphasized its importance in guiding further research aimed at identifying subgroups of liver cancer, such as those with less aggressive tumors, characterized by excellent overall survival and potentially obviating the need for additional treatments.

The blood‐based MRD assay not only predicts cancer recurrence and prognosis but also enhances postoperative management for HCC patients. Studies [35, 45, 46] showed that MRD‐negative patients derived limited benefit from adjuvant therapy, whereas MRD‐positive patients benefited significantly, with ctDNA clearance after adjuvant therapy linked to improved disease‐free survival. This underscores MRD as a vital biomarker for identifying patients who may benefit from adjuvant therapy and assessing treatment efficacy. In the era of combined immune checkpoint inhibitors and tyrosine kinase inhibitors for HCC [47], where response prediction and efficacy evaluation remain challenging, SeekInCure effectively meets these critical needs.

Our study also included two limitations. First, despite being a prospective study, the sample size remained relatively small and primarily focused on HBV‐positive HCC patients. Future studies should include HCC patients with other etiologies, such as HCV, metabolic, and autoimmune liver diseases, to enhance the applicability of SeekInCure. Second, longitudinal monitoring of ctDNA was lacking, particularly following the completion of cancer adjuvant treatment in MRD+ patients. Such monitoring could provide valuable insights into identifying responsive patients and evaluating the predictive value of ctDNA clearance post treatment. Nevertheless, this prospective study highlights the potential of blood‐based MRD status in predicting prognosis for early‐stage liver cancer patients.

## Author Contributions

**Qingqi Ren:** data curation (lead), resources (supporting). **Shiyong Li:** methodology (lead), visualization (equal), writing – original draft (equal). **Guolin Zhong:** investigation (equal), project administration (lead), writing – review and editing (equal). **Yunfei Li:** data curation (equal), formal analysis (equal). **Dao‐Ling Huang:** writing – original draft (equal). **Liangliang Zhang:** project administration (supporting), writing – original draft (supporting). **Yumin Feng:** methodology (equal). **Guanghui Long:** resources (supporting), supervision (supporting). **Mao Mao:** resources (lead), supervision (lead), writing – review and editing (lead).

## Conflicts of Interest

Shiyong Li and Mao Mao are full‐time employees and stock shareholders of SeekIn. Guolin Zhong, Yunfei Li, Dao‐Ling Huang, and Yumin Feng were full‐time employees of SeekIn. Guolin Zhong, Yunfei Li, and Yumin Feng are also the stock shareholders of SeekIn. Liangliang Zhang was a full‐time employee of Shenyou Bio, a wholly owned subsidiary of SeekIn. No other potential conflicts of interest were reported.

## Supporting information


**Figure S1.** Performance of updating FS method and published FS method in liver cancer detection. (A) Shallow whole genome sequencing was utilized to infer the global size distribution of cfDNA fragments from patient P09 pre‐surgery blood samples. The gray region represented the central distribution (the 25th and 75th percentiles) of fragment size frequencies obtained from 247 healthy individuals, and the black line depicted the mean value of fragment size across the healthy individuals. (B) Regional (5‐Mb) short‐to‐long ratio in patient P09 pre‐surgery blood samples. The gray line represents the background distribution of regional short‐to‐long ratios, calculated as the mean value across 5‐Mb regions among 247 healthy individuals. (C) Comparison of liver cancer detection performance between the updating FS method (FS.new) and the previously published FS method (FS.pub).

## Data Availability

We uploaded the clinical data information into the National GenBank Data Center in China (https://ngdc.cncb.ac.cn/gsub/submit/bioproject/PRJCA022312) with accession number PRJCA022312.
