# Supplementary material for: Non‐Invasive Tumor‐Naïve Minimal Residual Disease Detection of Liver Cancer by Incorporating Circulating Tumor DNA Features and Alpha‐Fetoprotein: A Prospective Study
Source: Cancer Med. 2024 Dec 20;13(24):e70511. doi: 10.1002/cam4.70511 (PMC11660314; doi:10.1002/cam4.70511)
Supplement: Supplementary file 1 — Figure S1. Performance of updating FS method and published FS method in liver cancer detection. (A) Shallow whole genome sequencing was utilized to infer the global size distribution of cfDNA fragments from patient P09 pre‐surgery blood samples. The gray region represented the central distribution (the 25th and 75th percentiles) of fragment size frequencies obtained from 247 healthy individuals, and the black line depicted the mean value of fragment size across the healthy individuals. (B) Regional (5‐Mb) short‐to‐long ratio in patient P09 pre‐surgery blood samples. The gray line represents the background distribution of regional short‐to‐long ratios, calculated as the mean value across 5‐Mb regions among 247 healthy individuals. (C) Comparison of liver cancer detection performance between the updating FS method (FS.new) and the previously published FS method (FS.pub). [file CAM4-13-e70511-s001.docx]

**Non-invasive Tumor-naïve Minimal Residual Disease Detection of** **Liver Cancer by Incorporating Circulating Tumor DNA Features and** **Alpha-fetoprotein: a Prospective Study**

Qingqi Ren^1^, Shiyong Li^2^, Guolin Zhong^2^, Yunfei Li^2^, Dao-Ling Huang^2^, Liangliang Zhang^3^, Yumin Feng^2^, Guanghui Long^1^, Mao Mao^4,5^

**Affiliations:**

^1^Department of Hepatopancreatobiliary Surgery, Peking University Shenzhen Hospital, Shenzhen 518000, China

^2^Research & Development, SeekIn Inc., Shenzhen 518000, China

^3^Clinical Laboratories, Shenyou Bio, Zhengzhou, 450000, China

^4^Research & Development, SeekIn Inc., San Diego, CA 92121, USA

^5^Yonsei Song-Dang Institute for Cancer Research, Yonsei University, Seoul 03722, Korea

**Corresponding author:**

Mao Mao, MD, PhD, Research & Development, SeekIn Inc., 10320 Camino Santa Fe, Suite G, San Diego, CA 92121, USA; Yonsei Song-Dang Institute for Cancer Research, Yonsei University, Seoul 03722, Korea; E-mail address: [maomao@yuhs.ac](mailto:maomao@yuhs.ac).

**Running title:** Multi-omics assay for liver cancer MRD detection

**Keywords:** liver cancer; MRD; AFP; sWGS; CNA; fragment size

**Supplementary Figures**

**Figure S1:** **Performance of updating FS method and published FS method in liver cancer detection.** (A) Shallow whole genome sequencing was utilized to infer the global size distribution of cfDNA fragments from patient P09 pre-surgery blood samples. The grey region represented the central distribution (the 25th and 75th percentiles) of fragment size frequencies obtained from 247 healthy individuals, and the black line depicted the mean value of fragment size across the healthy individuals. (B) Regional (5-Mb) short-to-long ratio in patient P09 pre-surgery blood samples. The grey line represents the background distribution of regional short-to-long ratios, calculated as the mean value across 5-Mb regions among 247 healthy individuals. (C) Comparison of liver cancer detection performance between the updating FS method (FS.new) and the previously published FS method (FS.pub).
